# Do Nonsteroidal Anti-Inflammatory Drugs Cause Endoprosthetic Loosening? Mid- to Long-Term Follow-Up of 100 Total Hip Arthroplasties after Local NSAID Infiltration

**DOI:** 10.1155/2015/703071

**Published:** 2015-05-18

**Authors:** Ikram Nizam, Lawrence Kohan, Clarice Field, Dennis Kerr

**Affiliations:** Joint Orthopaedic Centre, P.O. Box 240, Bondi Junction, NSW 1335, Australia

## Abstract

We evaluated the effect of local infiltration of NSAIDs on prosthetic fixation at mid- to long-term follow-up of total hip arthroplasties. Intra-articular local NSAID (ketorolac) was injected into hip joints and surrounding tissues intraoperatively and postoperatively as a part of multimodal pain management protocol. Clinical and radiographic evaluation was performed for any evidence of component loosening or failure and clinical outcomes in 100 total hip joint arthroplasties with a mean follow-up of 7.3 years (4.9 to 11 yrs). Radiographic analysis at the most recent follow-up showed no evidence of loosening, subsidence, or migration and no evidence of impending failure. Clinical outcomes showed improved Harris hip scores. Intra-articular NSAID used in the intraoperative/postoperative period in hip arthroplasty showed no evidence of prosthetic loosening at mid- to long-term follow-up.

## 1. Introduction 

Pain relief plays an important role in recovery from surgery and anaesthesia after total joint arthroplasty.

Since the late 90s, our team have been using an effective multimodal anaesthetic technique to improve quality and rate of recovery and enable early mobilization after total joint replacements [1]. This involved injecting local NSAID in a cocktail mixture with ropivacaine and adrenaline in and around the joint and as a result the implants “soaked” in this mixture.

Recently there have been reports that nonsteroidal anti-inflammatory drugs (NSAIDs) following total hip arthroplasty may have the potential to adversely affect endoprosthetic fixation, thereby increasing revision rates [2, 3].

Animal studies have suggested that NSAIDs impair fracture healing [4–10] and clinical studies have emerged stating that NSAIDs inhibit spinal fusion [11, 12] and retard healing of femoral diaphyseal fractures [13]. Other clinical and randomised control studies have implicated NSAIDs in the prevention of heterotopic ossification (HO) suggesting they may have an effect on bone metabolism and formation of new bone [14–19].

With our local infiltration technique becoming more popular, concerns have arisen regarding the use of NSAID in a joint replacement and whether short term local use does actually inhibit bone ingrowth or cause endoprosthetic loosening in the long run.

For this reason we analysed the radiographic and clinical outcomes of 100 consecutive total hip arthroplasties by a single surgeon at mid- to long-term follow-up to determine the incidence of periprosthetic loosening or failure in patients receiving infiltration of intraoperative and early postoperative NSAID.

## 2. Patients and Methods

Patients with symptoms of hip pain or stiffness due to end stage arthrosis/inflammatory arthritis, patients with osteonecrosis of the femoral head or poor bone mineral density who were not candidates for hip resurfacing, and those patients with fracture neck of femur after hip resurfacings were included in the study.

All patients were assessed and managed by a single surgeon and anaesthetist pre/intra/postoperatively.

We used a metal on metal combination of Birmingham resurfacing acetabular component, Birmingham modular heads, and various stems (cemented and uncemented).

All total consecutive hip joint arthroplasties were performed between December 1996 and January 2006. The files were retrieved from the joint reconstruction database where prospective information was entered. A total of 94 patients were included in the study.

Patients who had contraindications for the use of NSAIDs were not given NSAIDs and were excluded from the study.

Detailed informed consent was obtained before surgery. The study was approved by the local human research ethics committee.

Local anaesthetic mixture (RKA) contained 2.0 mg/mL of ropivacaine hydrochloride (Naropin, AstraZeneca Pty Ltd.) along with a standard 30 mg dose of ketorolac tromethamine (Toradol, Roche Products Pty Ltd.)  and 5–10 *μ*g/mL adrenaline. 150 to 200 mL was used to deliver the drug mixture reliably throughout the surgical field.

A total number of 100 metal on metal total hip arthroplasties were included in the study in 94 patients. There were 53 females and 41 males with an average age of 69.9, age ranging from 47 to 88 (standard deviation of ±7.9). The average BMI was 28.8 (range from 18.7 to 40.9 SD ± 12.9).

Of the 100 hips, 94 were primary hybrid hip arthroplasties; 6 patients had bilateral consecutive hip arthroplasties. Six hips were revisions to total hip arthroplasties, 5 of which were after failed hip resurfacings due to fracture of femoral necks.

Of the primary arthroplasties, in 90 patients, the indication for operation was end stage osteoarthritis and 4 had rheumatoid arthritis. Sixty-five femoral components were uncemented and thirty-five were cemented.

Comorbidities included 4 patients with non-insulin-dependent diabetes, 2 with mild renal failure, and 8 with stable cardiac problems and one patient was morbidly obese.

No patients were lost to follow-up, and 2 patients died (2 yrs and 4.2 yrs after procedure) where the death was not related to surgery.

## 3. Surgical Technique

A standard posterior approach was utilized for all hip arthroplasties, the acetabulum reamed, and the appropriate uncemented cup seated.

Up to 50 mls of the injection mixture previously described by our authors [1] was then injected into the tissues around the rim of the acetabulum, focusing both on the joint capsule and around the exposed gluteal and adductor muscles.

The femur was then prepared in the usual manner and the femoral component was inserted (cementless or uncemented).

A second injection was then made into the external rotators, gluteus tendon, and iliotibial band. The third injection was made to the skin and subcutaneous tissues.

A catheter was placed in the anterosuperior aspect of the hip joint. 10–15 mls of RKA mixture was injected immediately after wound closure to flood the joint.

**Figure 1:**
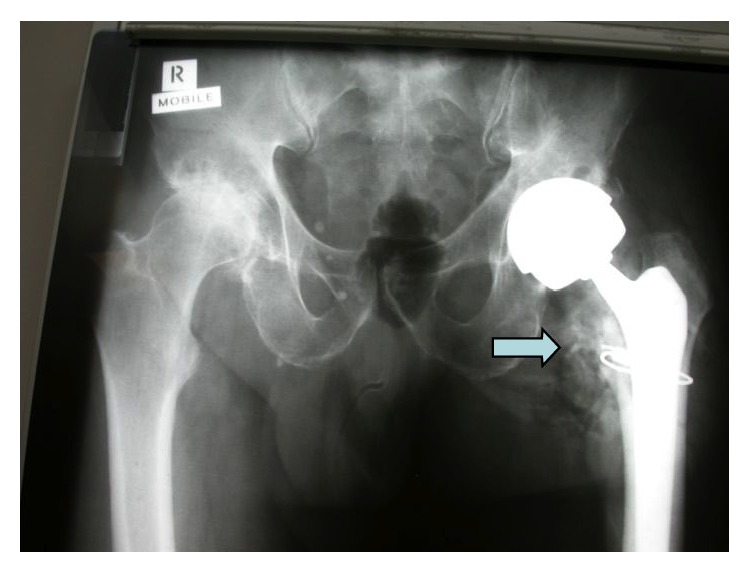
Distribution of local anaesthetic mixture with NSAID into the hip joint after insertion of prosthesis highlighted by injection of radiopaque agent (arrow), “soaking” the implants.

Figure 1 demonstrates the distribution of the mixture in the hip joint when injected with radiopaque agent.

No drains were used in any of the hip arthroplasties. A compression hip dressing was applied to all patients after wound closure.

All patients had oral Ibuprofen 400 mg given Post po for 24 hrs then as required over the next week and then discontinued. Oral analgesics, usually paracetamol 1 g combined with tramadol 50−100 mg or codeine 32 to 64 mg, were provided for use not more than 4 hourly as required. Patients were instructed to cease the tramadol or codeine and take only paracetamol as soon as the pain had decreased to an acceptable level. Aspirin 300 mg was given daily for 6 weeks for thromboprophylaxis unless the patient had previous history of deep venous thrombosis or pulmonary embolism. Then thromboprophylaxis was used with enoxaparin initially followed by warfarin.

## 4. Postoperative

All patients were mobilized approximately 4 hours after arthroplasty and thereafter every 2-3 hours during the day for a minimum of 25 meters.

Top-ups were performed at 15–20 hours postoperatively through the catheter with up to 50 mls of RKA mixture, the last 30 mls injected as the catheter was withdrawn.

Plain radiographs were performed on postoperative day one, which included an AP radiograph of the pelvis and a lateral radiograph.

Majority of patients were discharged in the first 24 hours after surgery with follow-up at home by phone, the following day.

## 5. Radiographic Analysis

We divided the periacetabular region as per DeLee Charnley zones to predict acetabular component loosening [20]. We defined a lucent zone on the radiograph as a dark line of demarcation around the acetabular component. The widths of these demarcations were divided into 2 groups, group one where the width was less than 1 mm and group 2 where the width was more than 1 mm. In our study we classified the uncemented cup as loose if all three zones had less than 1 mm lucency or 2 or more zones had more than 1 mm lucency. The demarcation was measured on the plain films without correction for the 10 percent of magnification. Any cup migration or tilting was also recorded.

We used a modified system adopted by Persson et al. [2] to determine femoral component loosening.

The components of the total hip arthroplasty and hence the THR were considered radiographically loose if one or both components fulfilled one or more of the above criteria.

The radiographic evaluation was carried out by an Orthopaedic Fellow with good experience in radiological evaluation of hip arthroplasties, who was not a part of the operating team.

Survivorship was determined using the Kaplan-Meier method and confidence intervals were calculated to the 95% level and statistical significance was determined at* p* equal to or less than .05.

## 6. Results

### 6.1. Radiographic Results

**Figure 2:**
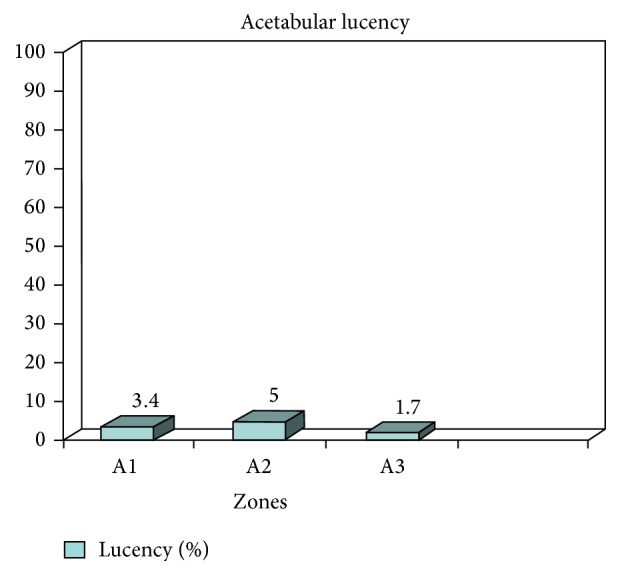
Showing percentage acetabular lucency in DeLee Charnley zones A1–A3. Majority of lucencies were less than 0.5 mm with no evidence of acetabular component loosening.

Acetabular component lucencies were minimal on radiographic analysis at the most recent follow-up 3.4% in zone 1, 5% in zone 2, and 1.7% in zone 3 (Figure 2).

**Figure 3:**
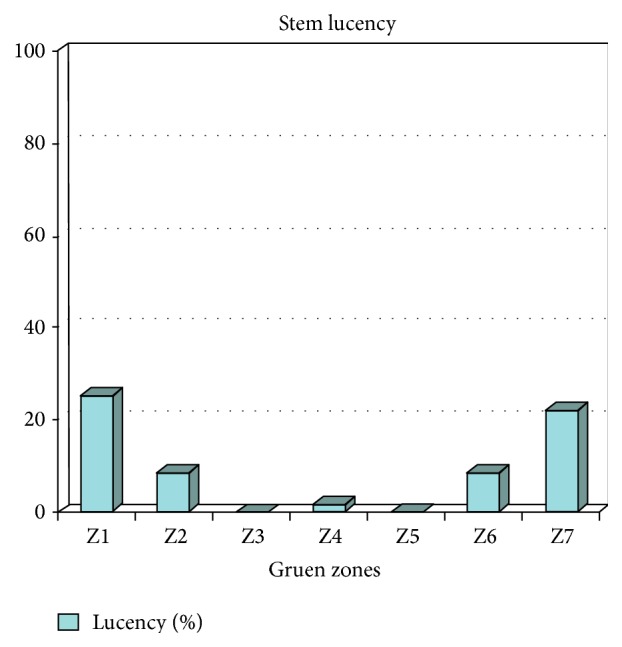
Showing lucent zones in the Gruen zones. >80% lucencies were less than 0.5 mm with no evidence of stem loosening.

Femoral lucency in the respective zones from 1 to 7 was 25%, 8.5%, 0%, 1.7%, 0%, 8.5%, and 22% (Figure 3).

Most lucencies (>80%) around the acetabular and femoral components were in group 1, <1 mm. No cases were found to have 2 or more contiguous lucent zones around acetabular components and no cases showed 3 or more contiguous lucent zones around the femoral components. We observed a zero incidence of lucent zones more than 2 mm around either femoral or acetabular components.

There was very minimal progression of radiological lucency from early postoperative period to the most recent follow-up in individual zones around the components.

No component migration or subsidence was noted and no components were revised for loosening and no impending failure was noted at the most recent follow-up.

### 6.2. Clinical Results

**Figure 4:**
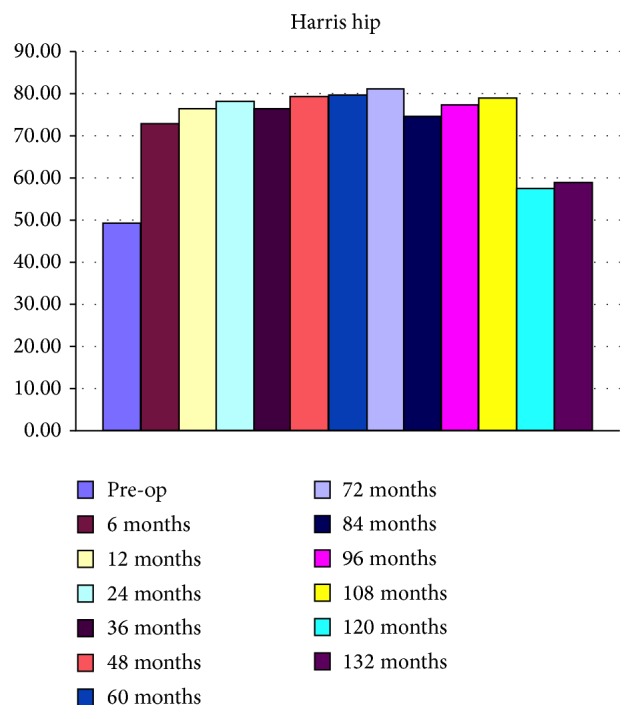
Harris hip scores showing significant improvements postoperatively from 6 months to 108 months and modest improvements from 108 to 132 months.

Clinical outcomes were measured in the form of Harris hip scores (HHS). Preoperative and postoperative scores achieved at follow-up are shown in Figure 4.

The Harris hip scores revealed statistically significant improvements (*p* < 0.05) postoperatively especially from twelve months to one hundred and eight months where improvements were good to fair compared to pre-op scores. No clinical evidence of loosening was detected in any patients and no patients had dislocations until the most recent follow-up.

## 7. Complications

Two patients had intraoperative fractures of the proximal shaft of femur and one patient had a perforated femur intraoperatively whilst broaching; the former two patients had circlarge wiring of the femur and all three had protected weight bearing for 6 weeks; the fractures united clinically and radiographically by three months and the patients progressed well.

One had an above-knee deep vein thrombosis. No evidence of superficial or deep wound infections or clinical manifestations of pulmonary embolism were seen in any patients until the most recent follow-up. Two patients had lateral peroneal nerve palsies after surgery, both eventually resolved.

No hip dislocations were noted until the most recent follow-up and none of the patients have had any revision surgery for failure or hip pain.

## 8. Discussion

Although several studies show inhibitory effects of NSAIDs on fracture healing and bone regeneration [4–10], the use of NSAIDs is a matter of debate and concern. No clinical studies have been conducted looking at the long-term effects of large volumes of intra-articular local infiltration of NSAID in the intraoperative and early postoperative period in total hip joint arthroplasty and its effects on endoprosthetic fixation.

Whilst satisfactory pain control enables early mobilization, the main purpose of this form of pain relief was to improve quality of pain relief and recovery following joint arthroplasty with the intent of avoiding sedation and physiological disturbance in the early postoperative phase, reducing thromboembolic events, hospital bed stays, and possibly hospital acquired infections with NSAID as an important component of this pain management technique. This may not be achievable through conventional pain management modalities.

The therapeutic effect of NSAIDs is thought to be achieved by inhibition of prostaglandins (PGs) which, in turn, influences osteogenesis, angiogenesis, fracture healing, and remodelling of bone. Experimental data suggests that NSAIDs delay fracture healing [4–10] and may inhibit bone ingrowth in porous implants [3, 21] reducing shear strength between implants and possibly increasing likelihood of loosening and subsequent failure. Another study looking at bone ingrowth in porous coated implants in canine models given indomethacin 2 weeks prior to surgery and up to 18 weeks after surgery showed no difference between treated or control groups in bone-implant interface shear strength or bone ingrowth [22].

Our study showed no evidence of radiological loosening at an average of 7.3 years (median 7.1 years) follow-up with no features suggestive of component subsidence, migration, or impending failure and a component survivorship of 100% in the remaining 98 patients. In contrast Kerr and Kohan [1] reported a high percentage of hip arthroplasty revisions in the NSAID treated groups compared to placebo group. The prostheses used were all cemented femoral components (Charnley or Exeter) where bone ingrowth into femoral components is not possible. Our study had a majority of patients (65%) with uncemented femoral components with radiographic evidence of good bone-implant interface with no signs of femoral component loosening or failure.

Femoral broaching may induce “microfractures” in the femoral endosteal surface. Although experimental and clinical studies state that early regenerative phase of bone healing after trauma is sensitive to NSAID [9, 10, 17, 18], our study revealed no evidence of radiological or clinical failure at the bone-implant or cement-bone interface at subsequent follow-up.

Clinical evaluation revealed improvements in individual HHS with no complaints of hip pain suggesting loosening or impending failure. There were significant improvements in the HHS until 108 months after surgery but this declined slightly towards the 120- and 132-month mark. This may be due to reduction in functional levels as they were almost 10-11 years after surgery (average age at the time being > 80 years and majority of patients being females).

The patients with intraoperative proximal femoral fractures in this study despite having the normal NSAID dose infiltrated also healed in the appropriate time with no adverse outcomes and so did the patients with rheumatoid arthritis who were on steroids preoperatively. We did not regard inflammatory arthritis as a contraindication to NSAID infiltration in arthroplasty although patient and dose selection was varied in the presence of renal impairment.

We conclude that NSAID infiltration as a part of the RKA mixture in intraoperative and early postoperative period is safe and useful in total joint arthroplasty.

Limitations of the study include a small population size and the inability to randomize patients. Our aim was to conduct a randomized controlled double blinded study to determine the success of local anaesthetic mixture in joint arthroplasty and subsequently the effects of short-term use of NSAIDs on prosthetic fixation; however, the patients were reluctant to take part in the study as they were aware of the possibility of slower and poorer quality of recovery if they were selected into the control group.

The AP radiographs may have rotational errors of the component when interpreting lucent zones, but we minimized this by performing radiographs through a single private institution with strict criteria for AP films.

## 9. Conclusions

The use of local infiltration NSAIDs in the intraoperative and early postoperative phase was not associated with endoprosthetic loosening in mid- to long-term follow-up in this series of hip joint arthroplasties. Prolonged NSAID usage postoperatively should be approached with caution.
